# Direct Use of a Saliva-Collected Cotton Swab in Lateral Flow Immunoassay for the Detection of Cotinine

**DOI:** 10.3390/bios12040214

**Published:** 2022-04-06

**Authors:** Chaewon Jung, Mingon Kim

**Affiliations:** Department of Chemistry, School of Physics and Chemistry, Gwangju Institute of Science and Technology, Gwangju 61005, Korea; jcw4444@gist.ac.kr

**Keywords:** lateral flow immunoassay, on-site salivary test, cotinine detection, point of care, smoking test, saliva test

## Abstract

The detection of salivary cotinine is useful for convenient smoking tests in spite of the high background effect of saliva. For precise results, the conventional salivary cotinine analysis for smoking detection requires complex pretreatment processes. Hence, in this study, we developed a modified paper-based lateral flow immunoassay (LFIA), termed “gap-LFIA”, for the direct application of saliva collected using cotton swabs for on-site detection. The gap-LFIA was constructed by modifying a conventional LFIA sensor, where the sample pad was divided to have a 3 mm gap. A saliva-collected cotton swab was inserted into the gap, and then, a buffer solution was added to the outer sample pad to dilute the saliva automatically. The gap-LFIA reduced the interference in salivary samples and showed improved signals, allowing for using the whole saliva directly without additional steps. Further, the deviation of results using a strip was less than that when the saliva was not diluted in a conventional cotinine kit, and it helped to distinguish between smokers and non-smokers more clearly in 15 min. This method of automatic dilution may apply to various clinical samples, including blood and serum, for direct application in future detections.

## 1. Introduction

Smoking is a widespread social problem, and there are 1.1 billion smokers worldwide. According to a recent report by the World Health Organization, over 8 million people died from smoking in 2019 [1]. It is associated with various diseases such as cardiovascular diseases, cancer, diabetes, and lung diseases [2,3]. In the form of passive smoking, smoking also affects non-smokers, especially children and pregnant women [4]. Due to its negative effects, smoking in public places has been prohibited by law in many countries. Moreover, social awareness to encourage smoking cessation is emerging. Therefore, immediate monitoring and testing of smoking is essential not only to maintain public health but also to help smokers who already know the harmful effects and want to quit.

Cotinine, a biomarker to detect smoking, is the main metabolite of nicotine, a highly addictive substance in cigarettes. It is a good biomarker with a long half-life of about 18 h after smoking; it is produced by the liver, kidneys, and lungs within 1 to 2 h after smoking and can be detected in blood, saliva, and urine samples. Traditional cotinine detection methods typically include high-performance chromatography (HPLC) and gas chromatography-mass spectroscopy (GC-MS). Although these chromatographic methods have relatively high sensitivity and accuracy, they are time-consuming and require expensive analytic instruments and personnel training. Like these methods, enzyme-linked immunosorbent assay (ELISA), a commonly used method, is also used at laboratory scale and is not easily accessible to general users. (Table 1) [5,6,7,8,9].

For immediately on-site and portable cotinine detection, colorimetric lateral flow immunoassay (LFIA) is used with gold conjugates. Because the cotinine has an insufficient size for a common sandwich immunoassay, competitive substance and antibodies were immobilized on paper strips to detect small molecules by competitive immunoassay [10]. When cotinine is present in the sample, fewer antibodies bind to the immobilized competitive substance in the test line. As a sample for detecting cotinine, most studies use urine or saliva, which are non-invasive and more accessible than other biological liquids. Although saliva is more advantageous for testing than urine, which is limited in time and space for field diagnosis, most commercially available cotinine kits are urine-based, not saliva-based, because of the low sensitivity of kits using saliva with high viscosity and impurities [11,12,13]. The composition of saliva differs from person to person, and it depends on food intake, hydration status, smoking, and saliva collection method. Moreover, viscosity of mucin from saliva could affect downstream analysis [14]. Therefore, highly sensitive studies require oral hygiene procedures to avoid the contamination of samples by food. The pretreatments make it difficult to set up proper rapid saliva testing conditions applicable in on-site detection. Therefore, in the cases of commercial on-site saliva-based detection kits, they require filtering or dilution after collection of saliva by spitting or chewing sponge for using whole saliva, in order to overcome the background effect (Table 2). Moreover, they require more volume of saliva and time to collect a sufficient amount of sample for testing. In this study, a simpler and more accurate saliva-based detection method for cotinine that can harness the convenience of saliva is developed, using a cotton swab. Moreover, since a relatively identical amount of sample is applied using the swab, the individual differences between users can be reduced compared to conventional cotinine kits.

A cotton swab is a useful tool to collect a saliva sample, thus making it non-invasive, easy-to-handle, and better accessible for users. Further, it can acquire similar sample volumes without using quantitative instruments. Thus, many on-site detection kits include swabs to collect samples such as saliva, nasal fluid, or environmental samples. In many studies, cotton swabs have been used for sample collection, similar to nasal fluids for respiratory virus tests, but the samples collected by swabs require a resuspension step in another buffer to apply the detection process, without it being applied to the whole saliva.

**Figure 1:**
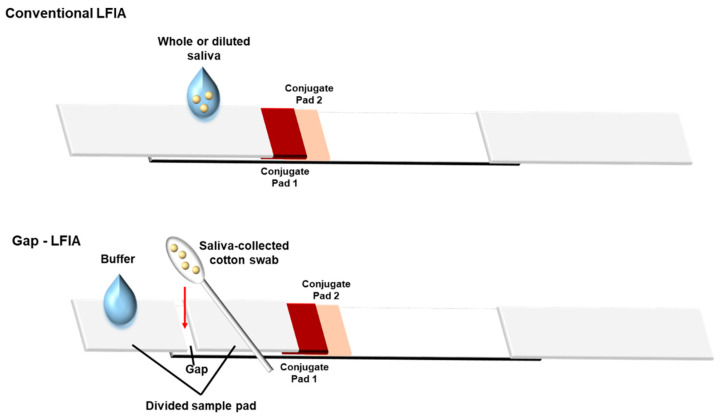
Structure of “gap-LFIA” with a saliva-collected cotton swab for detection of salivary cotinine compared to the conventional LFIA. The saliva is collected directly from the mouth and applied to the gap-LFIA. The cotton swab is inserted into the gap formed by the divided sample pads and allowed to connect.

Another advantage of this study is that it does not include an additional dilution step. The gap-LFIA is a simple device consisting of a sample pad having a 3 mm gap, modified from conventional LFIA strips, so that it can be applied directly to the sensor without dilution by using a cotton swab as a collection tool. The saliva-absorbed cotton swab can be easily inserted into the gap on the divided pad, and then, the addition of buffer solution on the sample pad dilutes saliva samples in the swab (Figure 1). The saliva does not flow all at once, but the buffer flows, and the saliva dissolves little by little. It reduces the possibility that the undiluted saliva flow first and the membrane be clogged with impurities by the viscous material in the saliva. In this study, the gap-LFIA was designed and tested as a salivary cotinine detection method for its rapid, convenient, and cost-effective use for smoking detection. Compared to the conventional LFIA with saliva, the method allows the direct use of the whole saliva collected using a cotton swab from mouth to be applied directly to a cotinine sensor. In addition, the accuracy was improved by reducing the deviation without additional preparation or equipment.

## 2. Materials and Methods

### 2.1. Chemicals and Instruments

Free cotinine, anti-mouse immunoglobulin G (IgG) antibody, and skim milk powder were purchased from Sigma-Aldrich (St. Louis, MO, USA). Gold nanoparticles (AuNPs) were purchased from BB International (Cardiff, UK). Anti-cotinine antibodies and cotinine-bovine serum albumin (BSA) were purchased from CalBioreagent (Foster City, CA, USA). Phosphate-buffered saline (20× PBS, pH 7.4) was obtained from Biosesang Co. (Seongnam, Korea). Borate buffer (20×, 1 M, pH 8.5) was purchased from Thermo Scientific (Waltham, MA, USA). Nitrocellulose membranes (FF80HP) were purchased from GE Healthcare (Seoul, Korea). Absorbent (Grade222), conjugate (Glass fiber 6613), and sample pads (Grade222) were purchased from Alshtrom Munksjö (Helsinki, Finland). A salivary cotinine quantitative enzyme immunoassay kit was purchased from Salimetrics. (Carlsbad, CA, USA). A microcentrifuge (Hanil, Kimpo, Korea) was used to separate free antibody and skim milk from the AuNP conjugates. A dispenser (DCI100, Zeta Co., Gunpo, Korea) was used to immobilize the cotinine-BSA and anti-cotinine antibodies on test and control lines of the FF80HP membrane. A drying oven (KO-100, LK Lab Co., Namyangju, Korea) was used to incubate the samples and to dry the membranes. A cutting device (TBC-50Ts, Taewoo Co., Namyangju, Korea) was used for making the test strips. All visible measurements were performed using a ChemiDoc XPS+ imaging system (Bio-Rad, Hercules, CA, USA) and analyzed with the Image Lab software (ver. 5.2.1) (Bio-Rad). The absorbance of ELISA was detected using Cytation 5 Cell Imaging Multi-Mode Reader (BioTek, Winooski, VT, USA).

### 2.2. Construction of Gold Conjugates with Antibody

An AuNP solution (40 nm in diameter, 1× AuNP, λmax optical density = 1.0) (1 mL) was added to 100 μL of 100 mM borate buffer (pH 8.5), to which 10 μg of the anti-mouse IgG antibodies was added. After incubation for 30 min at room temperature, 100 μL of 0.1% skim milk in 10 mM borate buffer (pH 8.5) was added to block the surface of AuNP conjugates. After incubation for 30 min, the conjugates were washed with 10 mM borate buffer (pH 8.5) three-times by centrifugation (6500 rpm, 20 min, 10 °C) and resuspended in a 50 μL storage buffer (5% trehalose, 0.5% skim milk, 0.2% Tween 20, and 1% Triton X-100 in 1× PBS) to maintain the affinity of the conjugates. The 20× AuNP conjugate stock was stored at 4 °C.

### 2.3. Preparation of Gap-LFIA Strip Sensor

For cotinine LFIA detection, 1 mg/mL of cotinine-BSA (test line) and 100 μg/mL anti-mouse IgG antibody (control line) were immobilized on the FF80HP membrane with 1 μL/cm of each solution. The distance between the test line and control line in the LFIA strip was approximately 5 mm. After immobilization, the membranes were oven-dried at 37 °C for 1 h. An absorbance pad was attached to the upper part of the membrane with a 3 mm overlap, was cut to 3.8 cm width using the cutting device, and was stored in a humidity-controlled chamber (21~23% humidity) before use. Conjugate pads (3.8 × 4.0 mm), pre-blocked with the storage buffer, were applied with 3.8 μL of the anti-cotinine antibody (conjugate pad 1) and AuNP-antibody conjugates (conjugate pad 2), both diluted with 1× PBS having 1% polyvinylpyrrolidone (PVP) and 0.5% Tween 20. The conjugate pads were dried and attached to the bottom of the strip with a 3 mm overlap. A sample pad, divided into two segments with a 3 mm gap for inserting the cotton swab, was placed further below as depicted. The gap-LFIA strip sensor was constructed immediately before the experiments.

### 2.4. Gap-LFIA Testing of Cotinine in a Buffer and Spiked Saliva

**Figure 2:**
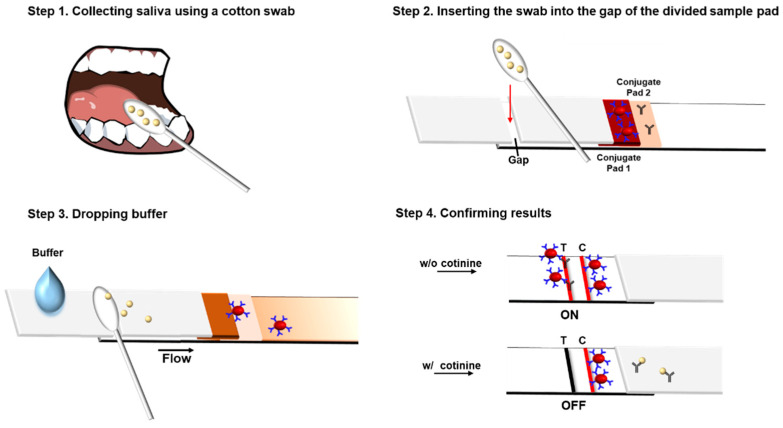
Procedure of the gap-LFIA system for salivary cotinine detection. C, Control line (anti-mouse IgG dried); T, Test line (cotinine-BSA dried). The intensity of test lines in Step 4 signal “on” without cotinine and “off” in the presence of cotinine.

A standard cotinine stock solution was serially diluted to 1, 10, and 100 ng/mL using 1× PBS and spiked at the same concentrations into real saliva. At least 120 μL of cotinine sample solution was taken to saturate each cotton swab, which was then inserted into the gap in the divided sample pads of the test strip. Then, the buffer (100 μL, 1× PBS containing 1% PVP and 0.5% Tween 20) was added to the bottom sample pad to flow along the strip through the cotton swab, carrying the sample toward the sensor region (Figure 2). The added buffer mixes with the saliva contained in the cotton swab and moves toward the membrane. The strip was incubated for 15 min at room temperature, and the intensities of the control and test lines were observed. All images were obtained using the ChemiDoc XPS+ imaging system and analyzed using the Image Lab 6.0 program. First, the various concentrations of the anti-cotinine antibody (12.5, 25, and 50 ng/mL) and 1 mg/mL of cotinine-BSA, which is sufficient for competition with free cotinine in the saliva samples, were tested. To determine the optimal concentration of the anti-cotinine antibody, the ratio of changes of control to test (C/T) line density was compared by analyzing cotinine concentrations.

### 2.5. Test of Real Samples Collected from Smokers and Non-Smokers and Comparison with ELISA

Human saliva samples for cotinine detection were obtained from 14 volunteers including 6 smokers, and all participants aged 20 to 50 gave informed consent to the study approved by the Gwangju Institute of Science and Technology. The amount of smoking in the last 24 h before saliva collection was recorded for each volunteer. The saliva samples were collected by absorbing several cotton swabs and spitting in snap tubes from the mouth of the volunteers without a hygiene procedure. The cotton swabs and saliva samples were stored in each snap tube at 4 °C, and the collected samples were used within 24 h for the gap-LFIA tests. For the verification of the gap-LFIA, the results were compared with the results of a commercial salivary cotinine ELISA kit using the same saliva samples. Further, in this study, high concentrations of caffeine and nicotine with cotinine were tested using the sensor to confirm its selectivity. Caffeine is an alkaloid that can often be found in human saliva, and nicotine is the precursor of cotinine with a similar structure [15].

## 3. Results and Discussion

### 3.1. Optimization of the Gap-LFIA Sensor for Cotinine Using Cotton Swabs

In this study, we developed the gap-LFIA, a modified cotinine LFIA sensor, to directly use for the whole saliva collected via a cotton swab. The detection of cotinine with the gap-LFIA is a competitive assay between salivary cotinine and cotinine-BSA immobilized on a detection membrane. To improve the cotinine detection sensitivity of the gap-LFIA strips, the concentrations of the anti-cotinine antibody and secondary antibody-AuNP conjugates dried on each conjugate pad were optimized. The conjugates required fewer primary antibodies and improved LFIA sensitivity.

For the gap-LFIA detection, the reproducibility of collecting saliva by cotton swabs was confirmed. The average volume of collected saliva was measured by repeated trials of the weight of the swab before and after saliva absorption (Figure S1A). Moreover, a portion of collected saliva involved in the detection from the cotton swab was obtained by measuring the absorbance of remaining pigment spiked in saliva before and after buffer addition. The cotton swab was saturated using 112 ± 10.1 μL of the buffer or 131 ± 8.5 μL of the saliva with approximately 56% of the saliva flowing into the membrane when 100 μL of the buffer was added, regardless of pigment concentrations (Figure S1B).

### 3.2. Sensitivity and Specificity of the LFIA Test for Standard Cotinine and Spiked Saliva

The cotton swabs saturated with the standard cotinine solutions of various concentrations were applied on the test strip. Free cotinine in the inserted cotton swab flowed to the membrane after the addition of 100 μL of the buffer on the outer sample pad and competed with cotinine-BSA immobilized on the test line. In low cotinine concentration samples, most of the primary cotinine antibodies on the conjugate pad reacted with the cotinine-BSA conjugates, and the residual antibodies were captured by the anti-mouse antibodies on the control line (signal “on”). Each primary antibody of the two lines was coupled to the secondary antibody-AuNP that generated a signal. Free cotinine in the cotton swabs reacted with the most anti-cotinine antibodies, and a signal with lower intensity was detected (signal “off”). Furthermore, 25 ng/mL anti-cotinine antibody in the conjugate pads was well-distinguished in lower cotinine concentrations (Figure S2), and lesser cotinine-BSA decreased the intensities of the test line. Various concentrations of the anti-mouse IgG-AuNP conjugates, dried on the conjugate pads, were also tested. The highly concentrated AuNPs, secondary antibody-conjugated, bind with the anti-cotinine antibodies and enhance the signal of each line. The amount of AuNPs did not affect the competitive immunoassay of cotinine. Cotinine-BSA as competitive substance was immobilized at a low concentration in the test line for sensitive detection of cotinine in saliva, and as shown in Figure S3, it was optimized to 5X to clearly “on” the signal of the test line in the control.

**Figure 3:**
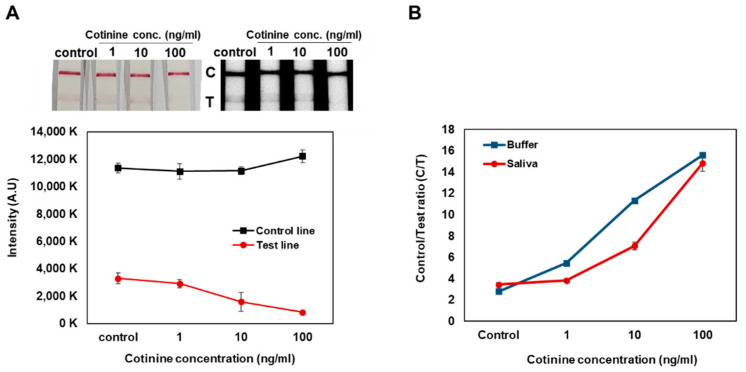
Cotinine concentration test spiked in saliva by the gap-LFIA with 25 ng/mL anti-cotinine antibody on conjugate pads. (**A**) Comparison for intensities of control line and test line. (**B**) Conversion to Control to Test ratio. All data shown are presented as the mean ± SD (*n* = 3).

By the gap-LFIA, the signal of test line for 1 ng/mL of cotinine in the buffer and saliva could be distinguished from that of the control, and a cotinine concentration dependent increase in C/T ratio in the samples was observed (Figure 3). The sensitivity of cotinine detection in saliva samples was slightly lower than in buffer, indicating that the saliva contained within the samples could affect immunoassay reaction conditions.

### 3.3. Salivary Cotinine Sensitivity Test with Smokers and Non-Smokers

**Figure 4:**
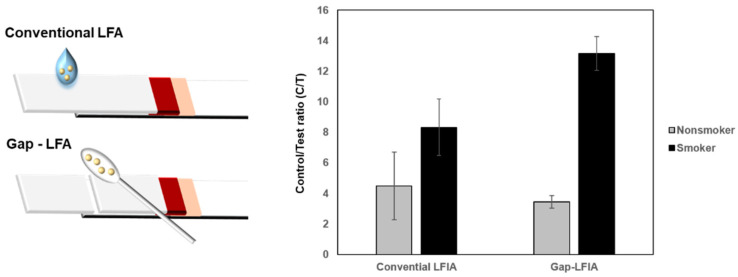
Comparison of C/T values of the conventional LFIA and gap-LFIA. All data shown are presented as the mean ± SD (*n* = 3).

The C/T values of the saliva samples from smokers and non-smokers showed distinct differences in the LFIA test. When the whole saliva was used without dilution, deviations in C/T values were higher compared with those of gap-LFIA. Automatically diluted saliva reduced background hindrance and provided appropriate conditions for cotinine detection (Figure 4). The results indicate that gap-LFIA is more accurate for immunoassay than conventional LFIA and is more suitable for saliva-based detection. In addition, unlike the existing method of directly applying whole saliva, the saliva is slowly drawn out by the buffer, so there is little chance that the flow will be blocked due to the components in the saliva in the paper-based strip. In the general LFIA, the average C/T value for non-smokers was 4.5 (±2.2) and 8.3 (±1.8) for smokers. In the general LFIA, the average C/T value for non-smokers was 4.5 (±2.2) and 8.3 (±1.8) for smokers. In contrast, it was 3.4 (±0.4) for non-smokers in gap-LFIA and 13.2 (±1.1) for smokers. When the same samples were applied to the gap-LFIA, it was confirmed that the difference between smokers and non-smokers was larger, and the deviation between groups decreased compared to the conventional LFIA. Most cotinine diagnostics have a cutoff of 10–100 ng/mL, which is usually in a wide range for detection but not as highly sensitive. The reason is that salivary cotinine is as high as a few ng/mL in non-smokers and 20 ng/mL or more in steady smokers. In this study, the C/T ratio was calculated and analyzed by testing up to the lowest cutoff of 10 ng/mL and lower concentrations. The C/T ratio of 10 ng/mL cotinine in saliva was about 7.1 (Figure 3B). The distinction between smokers and nonsmokers is ambiguous in C/T 7.1 due to the high standard deviation in conventional LFIA, but it can be seen that they are clearly distinguished in the gap-LFIA (Figure 4). Visually, at a cotinine concentration of 10 ng/mL, the signal of the test line was significantly weakened, showing a difference from the control line.

**Figure 5:**
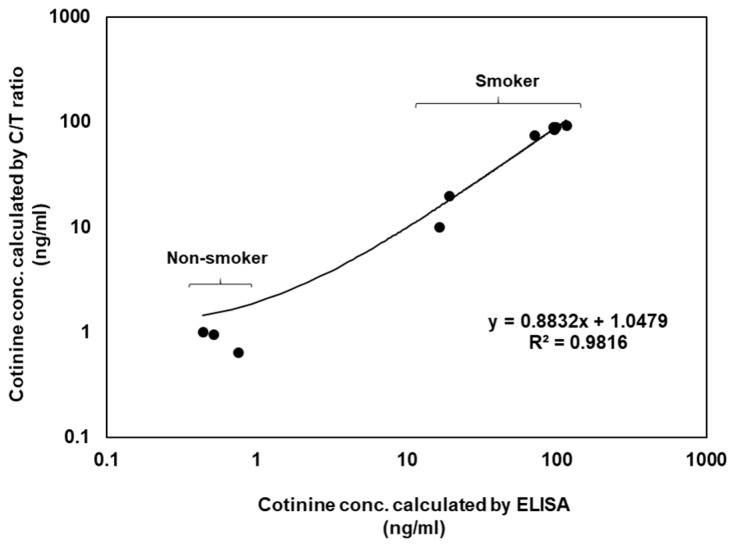
Correlation of cotinine concentrations calculated by ELISA and the gap-LFIA C/T ratios in salivary samples.

For the verification of this sensor, the results were compared with those of the conventional ELISA data. The comparative ELISA kit also used a competitive immunoassay for small molecule detection. ELISA kits have a cotinine detection range of 0.8–200 ng/mL, but this sensor had a 1–100 ng/mL cotinine detection range (Figure S4). Cotinine correlation curves (R^2^ = 0.9816) were calculated using the cotinine ELISA kit and the gap-LFIA test using the saliva samples from 14 volunteers (Figure 5). ELISA takes at least 2 h and has complicated experimental procedures with multiple steps. In contrast, the results of the LFIA test were ready in 15 min. The gap-LFIA has a similar detection range to ELISA, sufficient to distinguish between smokers and non-smokers, but is a simpler and faster method.

**Figure 6:**
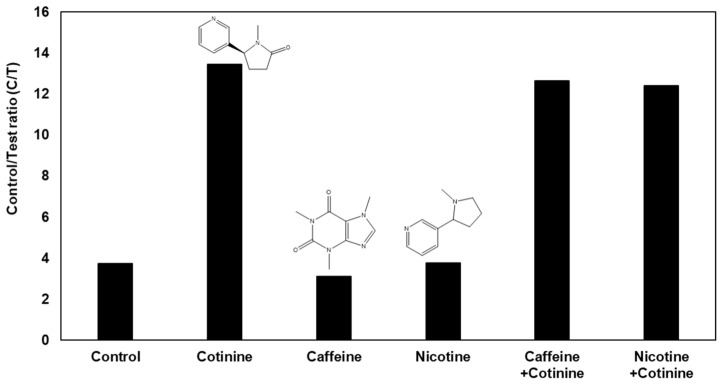
Selectivity test with 100 ng/mL cotinine along with 100-times caffeine and nicotine in the gap-LFIA.

The gap-LFIA sensor has selectivity for other compounds contained in saliva; hence, the compound does not affect the signal of the cotinine sensor. A selectivity test with a 100-fold concentration of caffeine and nicotine compared to that of cotinine, which have alkaloid structures in saliva, was performed (Figure 6). Regardless of the presence of caffeine or nicotine, the sensor produced the same result for cotinine and confirmed no interference.

## 4. Conclusions

Simple cotinine detection for smoking is important not only for individuals but also for public health. Although many cotinine assay kits have been commercialized, they involve various inconvenient processes to handle salivary samples. In this study, the gap-LFIA using cotton swab was shown as an easy-to-handle method for detecting cotinine from saliva. The gap-LFIA could apply the saliva directly to the strip without additional sample preparation steps and could dilute the saliva samples from the cotton swabs reducing background hindrance. As stated in the results, 1 ng/mL salivary cotinine was measured in 15 min by the image program. Moreover, the non-smokers and smokers could be more accurately distinguished, compared with the results obtained from the conventional LFIA without the gap. The results of gap-LFIA showed a high correlation with the conventional ELISA method, showing that it is reliable, fast, and easy to use for cotinine detection in saliva. In addition, the gap-LFIA can also be utilized for direct application of other different types of samples such as blood, seawater, or powder.

## Figures and Tables

**Table 1:** Traditional methods for the determination of salivary cotinine.

Detection Method	Collection	Pretreatment	Sensitivity	Reference
Microfluidic/ electrochemical	Cotton collector	Filter	1 pg/mL	[5]
LC-MS/MS	Spit	Centrifuge, dilution	1 pg/mL	[5]
Microfluidic immunofluorescence	Spit	Dilution	1 ng/mL	[6]
ELISA	n/a	n/a	1 ng/mL	[7]
Colorimetric immunoassay	Spit	Dilution	10 ng/mL	[8]
Colorimetric immunoassay	Cotton swab	n/a	10 ng/mL	This work

**Table 2:** Comparison of the gap-LFIA and commercialized saliva cotinine kits.

Company	Products	Collection Tool	Sensitivity
ConfirmBioscience	NicAlert™	Spit	10 ng/mL
NicoTests	NicoTests	Spit	30 ng/mL
ALCOPRO	iScreen Oral Fluid Nicotine Test	Sponge	30 ng/mL
STAT Technologies	NicDetect Oral Cotinine Test	Sponge	30 ng/mL
ALLTEST	Nico Quick Saliva Test	Stick	20 ng/mL
ONP	N-Checker	Spit	50 ng/mL
HUBIOTECH	NicoCheck Saliva Test	Stick	20 ng/mL

## Data Availability

Not applicable.

## Supplementary Materials

The following supporting information can be downloaded at https://www.mdpi.com/article/10.3390/bios12040214/s1. Figure S1: Reproducibility test of collecting and applying saliva using a cotton swab to the gap-LFIA. (**A**) Average sample volume collected by the cotton swab in Step 1 in Figure 2. All data shown are presented as the mean ± standard deviation (*n* = 7). (**B**) The portions of eluted saliva samples from the cotton swab in Step 3 in Figure 2. All data shown are presented as the mean ± standard deviation (*n* = 3). Figure S2: Optimization for the concentration of anti-cotinine antibody in the gap-LFIA. (**A**) 12.5 ng/mL, (**B**) 25 ng/mL, and (**C**) 50 ng/mL of the anti-cotinine antibody. Figure S3: Comparison of the intensity of the gap-LFIA with 25 ng/mL cotinine antibody by the concentration of AuNP-secondary Ab conjugates without cotinine. Figure S4: Cotinine ELISA test by spiked in saliva in the cotinine concentration range of smokers and non-smokers.
