# Supplementary material for: Direct Use of a Saliva-Collected Cotton Swab in Lateral Flow Immunoassay for the Detection of Cotinine
Source: Biosensors (Basel). 2022 Apr 6;12(4):214. doi: 10.3390/bios12040214 (PMC9029424; doi:10.3390/bios12040214)
Supplement: Supplementary file 1 [file biosensors-12-00214-s001.zip › biosensors-1593811-supplementary.pdf]

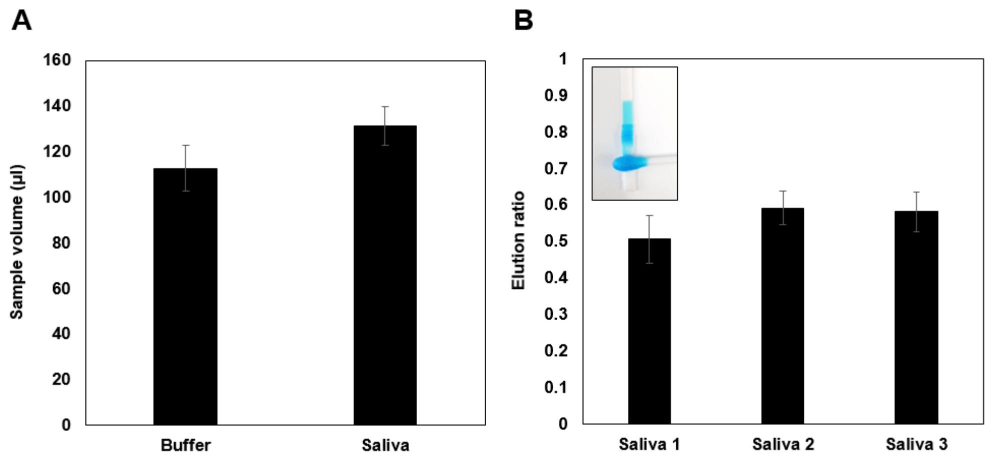

Figure S1. Reproducibility test of collecting and applying saliva using a cotton swab to the gap-LFIA. (**A**), Average sample volume collected by the cotton swab in Step 1 in Figure 2. All data shown are presented as the mean ± standard deviation (n = 7). (**B**), The portions of eluted saliva samples from the cotton swab in Step 3 in Figure 2. All data shown are presented as the mean ± standard deviation (n = 3).

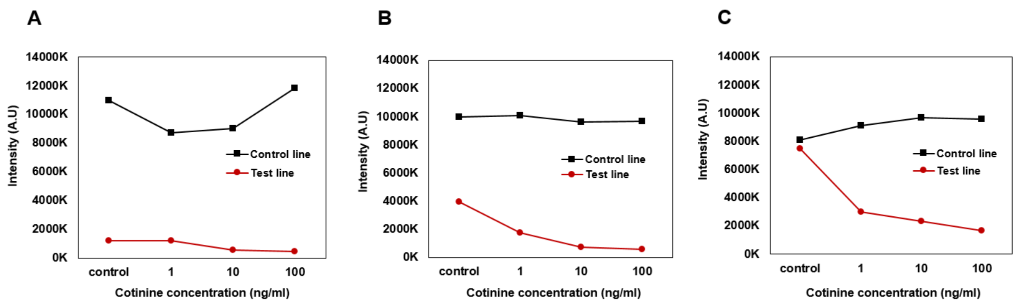

Figure S2. Optimization for the concentration of anti-cotinine antibody in the gap-LFIA. (**A**) 12.5 ng/ml, (**B**) 25 ng/ml, and (**C**) 50 ng/ml of the anti-cotinine antibody.

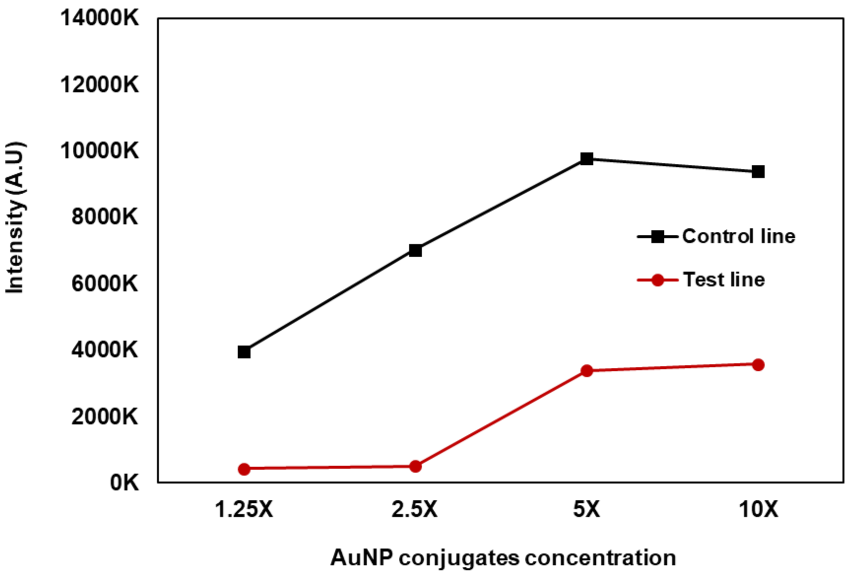

Figure S3. Comparison of the intensity of the gap-LFIA with 25 ng/mL cotinine antibody by the concentration of AuNP-secondary Ab conjugates without cotinine.

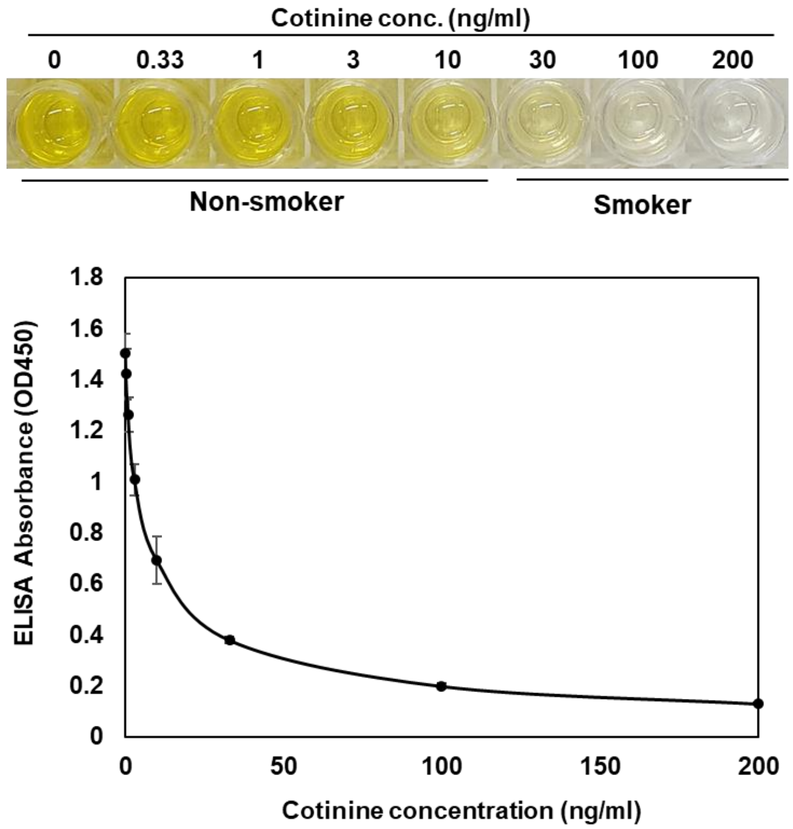

Figure S4. Cotinine ELISA test by spiked in saliva in the cotinine concentration range of smokers and non-smokers.
